# Supplementary material for: Transcriptomic Analyses of MYCN-Regulated Genes in Anaplastic Wilms’ Tumour Cell Lines Reveals Oncogenic Pathways and Potential Therapeutic Vulnerabilities
Source: Cancers (Basel). 2021 Feb 6;13(4):656. doi: 10.3390/cancers13040656 (PMC7915280; doi:10.3390/cancers13040656)
Supplement: Supplementary file 1 [file cancers-13-00656-s001.pdf]

cancers

MDPI

Supplementary Materials

Transcriptomic Analyses of MYCN-Regulated Genes in Anaplastic Wilms' Tumour Cell Lines Reveals Oncogenic Pathways and Potential Therapeutic Vulnerabilities

Marianna Szemes, Zsombor Melegh, Jacob Bellamy, Ji Hyun Park, Biyao Chen, Alexander Greenhough, Daniel Catchpoole and Karim Malik

Table S1. Oligonucleotides.

Targeted Gene	Name	Sequence (5′–3′)
ROBO1	F-ROBO1	ACAAGGCAGCAAAACGAGAC
	R-ROBO2	TGAGGAACTGGGATCTCTGG
CDKN1A	F-CDKN1A	TGGAGACTCTCAGGGTCGAAA
	R-CDKN1A	CGGCGTTTGGAGTGGTAGAA
LIN28B	F-LIN28B	GCCCCTTGGATATTCCAGTC
	R-LIN28B	TGACTCAAGGCCTTTGGAAC
NME1	F-NME1	CGGCCTGGTGAAATACATGC
	R-NME1	TCAGGGTGAAACCACAAGCC
MTAP	F-MTAP	CACAGCTTGTGGCTCCTTG
	R-MTAP	TGGCACAAGAATGACTTCCA
XPO5	F-XPO5	GCTCTGGATATGCTTGACGC
	R-XPO5	AACGCTGCATTCTTTCCAAG
DKC1	F-DKC1	TGCTGAAGTGGTAAAAGCCC
	R-DKC1	ATCAACTGAGGAGCTGCTGG
TOMM20	F-TOMM20	ACCGCAAAAGACGAAGTGAC
	R-TOMM20	AAAGCCCAGCTCTCTCCTTG
TIMM50	F-TIMM50	CACTATGCCCTGGAGGATGA
	R-TIMM50	GCCAAGGAAGAGGTTCTGCT
PDK1	F-PDK1	TCACGCTGGGTAATGAGGAT
	R-PDK1	ACACGAGGTCTTGGTGCAGT
WDR77	F-WDR77	GTGATGAGAATGGGACACGTCTC
	R-WDR77	CACAGCAAGTGTAGCAGTCTTC
PRMT1	F-PRMT1	GAGGCCGCGAACTGCATCAT
	R-PRMT1	TGGCTTTGACGATCTTCACC
REST	F-REST	TGGAAAATGCAACTATTTTTCAGA
	R-REST	CAAAGTTCACATTTATATGGGCG
STMN3	F-STMN3	CATGGCCAGCACCATTTC
	R-STMN3	TGGTAGACGGTATTGGGGTG
GDAP1	F-GDAP1	TTGGAGAAAGTCTTGGATCAGG
	R-GDAP1	CTGCCAGGGTGAAGGATTC
ENAH	F-ENAH	TGCTGGCCAGGAGGAGAAGAAT
	R-ENAH	ACTGGGCTGTGATAAGGGTGTGG

Table S2. Antibodies

Antibody	Catalog Number	Lot Number
MYCN	CST84406	na
b-actin-HRP	A3854	na
LIN28B	CST11965	1
TOMM20	11802-1-AP	na
PRMT1	CST2449	1
PRMT5	SC-376937	E2015
WDR77/MEP50	ab154190	YJ090522CS
ADMA	CST13522	1
SDMA	CST13222	4
REST	22242-1-AP	na

Table S3. Shared MYCN-regulated genes inWit49 and 17.94.

Genes	Wit49		17.94	
	log2(shrunkFC)	FDR	log2(shrunkFC)	FDR
RRBP1	2.228	0.00E+00	1.045	3.29E-21
ROBO1	1.776	6.46E-27	1.384	0.00E+00
HEG1	1.745	5.42E-28	1.207	2.99E-36
SIRT2	2.008	2.72E-35	0.714	1.69E-05
CCNG1	1.531	4.32E-31	1.188	3.05E-32
FAM3C	1.710	1.83E-35	0.960	1.92E-19
ERP44	1.493	4.26E-26	0.944	5.48E-19
PCDH8	1.780	5.36E-26	0.548	5.97E-07
CALU	1.499	1.28E-32	0.829	3.82E-23
FKBP14	1.501	5.27E-31	0.735	1.24E-09
TICAM2	1.429	1.22E-12	0.796	3.78E-07
ABR	1.361	1.10E-18	0.860	8.80E-15
CDKN1A	1.705	1.52E-18	0.491	4.05E-03
THBS1	1.530	2.93E-30	0.637	4.38E-09
TTC37	1.101	3.56E-17	0.996	8.60E-25
CAPN2	1.310	5.66E-27	0.776	2.46E-14
POLDIP2	1.301	6.33E-17	0.761	4.70E-11
PDGFC	1.265	5.88E-21	0.794	7.35E-10
CEP55	1.131	3.80E-15	0.926	1.96E-16
SV2A	1.211	3.93E-15	0.828	5.59E-13
LINC00467	1.068	2.16E-08	0.938	2.72E-10
CHSY1	1.238	7.83E-15	0.763	2.90E-09
GALNT10	1.325	1.01E-16	0.666	6.08E-07
MOSPD2	1.164	2.04E-11	0.804	1.79E-08
PNRC2	1.193	8.45E-21	0.731	6.58E-15
DCTN4	1.082	1.41E-14	0.838	9.16E-18
TMEM250	1.278	1.79E-16	0.622	1.67E-05
FN1	1.438	7.51E-24	0.444	2.41E-04
ALG14	1.340	1.01E-10	0.513	5.65E-03
USP12	1.196	1.31E-16	0.625	9.61E-07
MTPN	1.105	1.54E-16	0.709	1.21E-12
PDHX	1.082	7.15E-15	0.700	9.74E-07
ERO1B	0.828	5.70E-04	0.954	1.52E-11
PRR11	1.021	9.58E-11	0.747	5.16E-11

SEL1L	0.954	4.08E-10	0.810	5.39E-22
RAB23	1.098	3.29E-13	0.662	1.43E-08
ITPRIPL2	1.166	9.15E-15	0.592	5.51E-05
CASK	1.179	2.77E-15	0.577	2.26E-06
NHLRC3	1.089	1.45E-13	0.667	2.90E-08
MXRA5	1.130	1.03E-07	0.595	4.34E-05
RETREG2	1.101	4.64E-14	0.578	1.70E-06
DNAJB9	0.925	4.33E-08	0.734	8.37E-10
LYRM1	1.106	2.00E-08	0.552	2.03E-03
PURB	1.102	1.96E-16	0.553	5.11E-06
PRR5L	1.016	6.39E-06	0.637	2.21E-08
CELSR1	0.581	3.16E-02	1.062	5.87E-37
SEMA5A	1.232	1.79E-21	0.409	9.60E-06
RAD21	0.906	6.00E-09	0.727	5.29E-19
KITLG	1.070	2.49E-14	0.544	9.28E-06
FBN1	1.022	9.04E-10	0.577	6.13E-05
HIPK3	0.796	1.96E-09	0.794	1.56E-15
TEX2	1.152	5.74E-19	0.438	8.06E-03
CD164	0.958	3.91E-15	0.632	3.56E-13
LDLR	1.206	6.23E-18	0.380	5.72E-03
PIP5K1C	1.080	7.46E-08	0.495	1.74E-04
ERAP1	0.744	2.80E-06	0.825	2.86E-20
PM20D2	1.178	3.93E-12	0.388	4.23E-03
UNKL	1.070	2.07E-08	0.486	5.71E-03
GOLIM4	0.978	3.25E-08	0.557	4.37E-10
ZNF117	1.023	3.89E-07	0.503	5.39E-05
LAMP3	0.769	6.60E-03	0.754	6.08E-07
PCYT1B	1.001	1.71E-06	0.508	1.65E-03
SLC2A4RG	0.900	4.22E-07	0.610	1.29E-05
LUZP6	0.928	8.22E-06	0.573	1.04E-03
VANGL1	0.765	2.44E-05	0.731	8.77E-07
DBI	0.871	8.57E-09	0.598	4.63E-06
GNPTG	0.693	1.09E-03	0.752	1.18E-07
PPP2R5A	0.955	4.44E-09	0.485	5.64E-04
ITGA9	0.718	1.13E-02	0.716	4.63E-08
ATF2	0.667	8.21E-06	0.767	3.79E-13
CNOT3	0.908	5.90E-05	0.522	2.40E-05
ZBTB4	1.025	4.41E-10	0.403	4.91E-04
KPNA5	0.912	9.35E-10	0.506	8.97E-04
ALCAM	0.836	9.91E-09	0.577	1.82E-04
BAZ2A	0.956	1.69E-11	0.439	1.05E-05
COL1A1	0.596	5.09E-04	0.787	9.19E-10
KIAA0319L	0.823	3.85E-08	0.551	3.22E-08
REST	0.874	5.11E-08	0.497	2.33E-06
DCP1A	0.791	1.21E-05	0.574	2.76E-06
LYPLA1	0.847	1.25E-08	0.515	8.60E-06
CDKL5	0.871	1.02E-07	0.491	1.20E-03
YPEL4	0.759	3.26E-04	0.600	7.72E-05
NUCB2	0.855	3.46E-09	0.503	1.63E-06
MAGEL2	0.896	4.61E-04	0.458	1.40E-02
ZDHHC5	0.633	2.96E-05	0.706	4.05E-12

FOXN3	0.898	1.59E-06	0.438	2.39E-04
VLDLR	0.717	1.91E-03	0.616	6.41E-06
FICD	0.667	4.54E-03	0.662	3.12E-06
NAGA	0.683	6.67E-04	0.640	3.20E-07
ADCY6	0.872	1.04E-06	0.450	2.31E-03
SLC1A3	0.891	2.45E-07	0.425	4.41E-06
C5	0.856	4.06E-05	0.459	4.02E-03
MATN2	0.612	1.10E-05	0.701	1.06E-05
CREBRF	0.884	1.06E-07	0.429	1.26E-02
NRP2	0.782	4.53E-07	0.525	4.16E-03
MARCH8	0.799	7.68E-05	0.507	8.31E-04
HIPK2	0.860	1.05E-06	0.435	4.28E-05
CABLES2	0.796	3.12E-04	0.494	5.63E-04
SLC26A11	0.667	3.57E-04	0.622	1.33E-04
ORMDL1	0.669	4.61E-06	0.618	7.01E-08
ARMCX2	0.668	3.11E-04	0.616	7.78E-08
CAB39	0.709	2.47E-06	0.571	8.10E-07
FNDC3B	0.811	2.10E-09	0.465	1.61E-07
DENND6A	0.847	4.41E-09	0.428	3.84E-03
COL3A1	0.697	1.73E-05	0.578	4.87E-08
ADAMTS9	0.763	6.84E-03	0.509	6.65E-05
PLPP5	0.712	7.38E-05	0.549	1.44E-04
ZNF268	0.611	1.96E-04	0.650	3.16E-06
MAPK8	0.801	5.04E-06	0.459	2.87E-03
CDCA4	0.789	3.80E-06	0.464	1.38E-03
NCAM1	0.793	2.88E-07	0.457	1.01E-04
TNRC18	0.793	1.26E-06	0.450	3.71E-06
WIPI1	0.832	1.70E-06	0.407	2.71E-02
ZNF107	0.741	1.07E-04	0.497	5.35E-05
SUN2	0.848	9.72E-08	0.389	3.63E-04
ZNF217	0.773	1.05E-07	0.463	1.64E-04
BLOC1S6	0.596	4.27E-03	0.639	2.26E-07
TTBK2	0.697	5.68E-05	0.537	1.93E-04
TMEM127	0.851	1.62E-08	0.379	1.82E-03
TGFBR3	0.798	3.81E-06	0.407	1.22E-02
MEST	0.793	8.24E-07	0.407	6.17E-05
ZNF780A	0.767	9.98E-06	0.418	2.82E-02
AIDA	0.705	6.33E-05	0.464	1.54E-04
HAUS8	0.752	5.64E-05	0.414	1.47E-02
HHAT	0.724	2.99E-04	0.441	7.45E-03
ARID3A	0.727	7.79E-08	0.436	1.13E-05
FAM114A1	0.765	1.84E-05	0.388	1.46E-02
ECE1	0.753	2.18E-06	0.397	1.70E-04
DACT1	0.588	3.05E-02	0.555	2.01E-03
DTWD2	0.590	7.82E-03	0.553	1.89E-03
HELZ2	0.687	6.43E-05	0.455	1.50E-02
ZDHHC9	0.696	3.11E-04	0.444	1.68E-05
WDTC1	0.638	3.46E-04	0.500	3.24E-04
IQGAP2	0.679	2.35E-03	0.454	7.96E-04
RRM2B	0.648	1.96E-04	0.483	2.51E-03
RHBDD1	0.577	1.81E-02	0.552	1.82E-04

CREB3L2	0.722	1.99E-04	0.405	3.30E-05
FAM214B	0.628	3.77E-03	0.497	1.11E-04
HACD3	0.654	5.52E-06	0.469	2.51E-07
CGNL1	0.556	1.31E-02	0.562	1.82E-04
NUCKS1	0.693	6.97E-06	0.420	3.01E-06
TIMP3	0.590	2.14E-04	0.521	2.31E-04
AC099850.3	0.622	3.33E-02	0.481	1.21E-02
MAN2B2	0.696	6.67E-04	0.403	1.62E-02
EGFR	0.615	8.89E-05	0.485	9.95E-04
H6PD	0.569	6.04E-03	0.528	1.03E-04
LINC00963	0.570	4.81E-04	0.523	1.42E-04
AC008957.3	0.620	2.81E-02	0.470	1.47E-02
RECK	0.665	6.90E-03	0.423	2.71E-02
IKZF2	0.655	6.35E-03	0.430	3.30E-02
NKX3-2	0.668	1.68E-02	0.415	2.52E-02
BACE1	0.684	1.39E-04	0.396	2.35E-03
FRY	0.643	1.42E-05	0.432	1.16E-02
ST3GAL5	0.591	6.48E-03	0.483	6.94E-03
HYOU1	0.529	1.28E-04	0.544	3.08E-10
MAFG	0.647	1.62E-05	0.423	4.37E-03
MFSD6	0.655	2.62E-03	0.412	3.59E-02
PDIA4	0.546	1.15E-04	0.511	2.66E-08
MALAT1	0.609	2.41E-04	0.444	8.64E-05
ZNF70	0.628	1.46E-02	0.421	1.06E-02
GBA	0.622	1.91E-04	0.427	5.61E-04
USP40	0.425	2.30E-02	0.616	4.41E-07
PIK3CB	0.540	1.35E-02	0.497	4.77E-05
KCTD20	0.636	3.03E-05	0.399	6.55E-05
FAM213A	0.650	9.64E-06	0.384	1.42E-03
ETV5	0.635	1.75E-04	0.387	1.68E-03
ACOX1	0.591	3.47E-05	0.429	4.22E-03
ENTPD4	0.599	2.60E-04	0.409	5.39E-05
CKAP2L	0.552	1.54E-02	0.450	2.69E-04
CREM	0.618	3.32E-03	0.384	4.15E-02
AP003392.3	0.493	1.21E-02	0.506	1.30E-05
FAM129A	0.465	3.05E-02	0.532	5.79E-08
COL6A2	0.480	1.47E-02	0.517	1.94E-07
CRAT	0.542	1.29E-02	0.447	4.81E-03
PSD4	0.548	3.35E-02	0.437	2.56E-02
OGA	0.418	2.93E-02	0.564	5.38E-10
PRIMPOL	0.524	3.60E-02	0.454	1.76E-02
PPP1R2	0.533	8.60E-03	0.444	2.42E-03
ZFHX3	0.545	2.76E-03	0.430	1.82E-04
ZBED4	0.507	1.23E-02	0.464	1.79E-04
SLC4A7	0.564	2.92E-03	0.406	2.89E-04
CALR	0.460	1.53E-03	0.503	3.10E-09
AAK1	0.460	1.01E-02	0.496	4.65E-05
MFAP3L	0.493	4.13E-02	0.458	1.21E-02
SENP2	0.516	3.70E-03	0.433	1.64E-04
VEZF1	0.558	5.44E-04	0.387	4.52E-04
RSBN1	0.475	2.36E-02	0.470	2.27E-04

AC092069.1	0.463	1.55E-02	0.480	1.74E-05
HSPA13	0.552	4.07E-04	0.388	2.02E-04
NF1	0.488	1.05E-03	0.451	4.52E-05
H1F0	0.549	9.06E-04	0.387	4.81E-04
ERV3-1	0.546	1.65E-02	0.386	3.87E-02
DCHS1	0.536	1.73E-02	0.380	1.46E-03
CCPG1	0.500	1.57E-02	0.407	1.62E-03
CCDC90B	0.427	3.04E-02	0.473	1.60E-04
KBTBD6	0.485	1.30E-02	0.405	2.18E-02
FBXO38	0.481	3.55E-03	0.405	6.33E-03
TMED7-TICAM2	0.414	1.53E-02	0.464	1.97E-04
AGPAT3	0.490	1.05E-02	0.387	9.93E-04
ARMCX3	0.425	1.24E-02	0.443	4.37E-05
ATP2B4	0.383	4.22E-02	0.478	7.76E-07
OS9	0.451	3.37E-03	0.402	2.63E-05
STIL	0.432	2.83E-02	0.410	9.20E-04
MSMO1	0.444	2.26E-02	0.392	5.16E-03
NUCB1	0.419	1.48E-02	0.406	3.96E-05
TNRC6B	0.426	2.67E-02	0.397	5.39E-04
ARL2	0.429	3.15E-02	0.392	2.44E-02
FAM120AOS	0.440	8.39E-03	0.381	8.83E-03
STRADB	0.406	4.27E-02	0.401	1.89E-02
LIPA	0.356	3.79E-02	0.442	7.02E-04
ANXA7	0.381	2.00E-02	0.394	1.32E-02
PDIA3	0.352	2.68E-02	0.394	2.18E-05
HSPH1	-0.341	3.81E-02	-0.270	1.26E-02
RPL7	-0.352	3.92E-02	-0.266	1.37E-02
RSL1D1	-0.369	2.37E-02	-0.265	4.47E-02
ATP5PB	-0.343	4.55E-02	-0.293	7.36E-03
IMPDH2	-0.348	3.89E-02	-0.291	2.73E-02
PSMD12	-0.385	4.73E-02	-0.270	3.04E-02
GPS2	-0.373	3.76E-02	-0.288	1.71E-02
UBA2	-0.359	2.67E-02	-0.305	3.50E-03
UBE2N	-0.361	2.93E-02	-0.305	7.11E-03
CNOT8	-0.369	5.00E-02	-0.297	4.63E-02
CBX3	-0.370	2.56E-02	-0.303	2.10E-03
RPL36	-0.406	1.14E-02	-0.269	3.92E-02
AASDHPPT	-0.391	1.95E-02	-0.290	4.99E-02
NUDT21	-0.380	4.72E-02	-0.311	4.49E-03
SMARCC1	-0.336	4.70E-02	-0.364	8.11E-04
ZMPSTE24	-0.424	1.45E-02	-0.281	2.24E-02
YTHDF1	-0.412	1.87E-02	-0.297	2.67E-02
MRPS22	-0.372	4.47E-02	-0.336	4.08E-02
RPL6	-0.414	7.27E-03	-0.298	2.82E-03
PGAM1	-0.369	2.49E-02	-0.343	3.32E-03
MKRN2	-0.413	1.63E-02	-0.300	4.73E-02
DAXX	-0.361	4.93E-02	-0.357	2.37E-03
AGO2	-0.401	2.49E-02	-0.318	1.19E-02
EFTUD2	-0.368	3.07E-02	-0.356	9.68E-04
PHB2	-0.372	2.68E-02	-0.352	4.41E-04
EIF3M	-0.426	7.15E-03	-0.309	5.18E-03

MRPL9	-0.422	8.23E-03	-0.315	3.29E-02
BUD23	-0.422	2.35E-02	-0.319	1.00E-02
ANP32A	-0.438	2.68E-02	-0.304	1.01E-02
RFC3	-0.436	3.06E-02	-0.307	1.94E-02
MRPS15	-0.374	2.47E-02	-0.370	9.23E-03
UTP14A	-0.426	2.52E-02	-0.319	1.40E-02
QTRT2	-0.443	2.38E-02	-0.304	4.31E-02
EIF1AX	-0.441	7.05E-03	-0.307	2.97E-03
RBM8A	-0.461	1.14E-02	-0.290	1.40E-02
UMPS	-0.449	1.43E-02	-0.316	3.51E-02
AEBP1	-0.417	2.73E-02	-0.348	1.77E-03
AL136295.4	-0.374	3.95E-02	-0.392	2.39E-04
TIMM17A	-0.457	1.23E-02	-0.313	2.38E-02
DDX56	-0.495	1.32E-03	-0.278	2.98E-02
STK26	-0.356	4.88E-02	-0.418	5.98E-06
C8orf33	-0.391	3.43E-02	-0.384	8.22E-03
RUVBL1	-0.451	7.26E-03	-0.325	2.81E-03
CSE1L	-0.448	2.60E-03	-0.329	4.87E-04
NUP93	-0.392	2.30E-02	-0.385	6.04E-04
TUFM	-0.463	2.81E-03	-0.315	6.11E-03
SERBP1	-0.461	9.78E-04	-0.320	1.06E-03
SUPV3L1	-0.465	1.10E-02	-0.316	3.22E-02
TMEM123	-0.383	4.16E-02	-0.399	9.12E-05
PNPT1	-0.400	1.33E-02	-0.386	6.34E-03
TCP1	-0.442	3.56E-03	-0.345	4.70E-04
KNOP1	-0.414	2.70E-02	-0.377	1.22E-02
ZMAT2	-0.423	2.35E-02	-0.368	5.38E-03
RLIM	-0.430	5.48E-03	-0.369	1.82E-03
DHX15	-0.457	6.64E-03	-0.342	2.52E-04
CCDC6	-0.444	1.31E-02	-0.356	1.31E-02
PSMD10	-0.514	1.70E-03	-0.287	1.10E-02
IPO4	-0.383	3.48E-02	-0.419	7.60E-05
NOB1	-0.487	3.65E-02	-0.317	4.12E-02
E2F5	-0.403	2.50E-02	-0.401	2.27E-04
FARSB	-0.503	2.68E-03	-0.304	7.55E-03
RRP12	-0.386	3.79E-02	-0.423	7.55E-04
MYL12B	-0.458	2.08E-03	-0.357	2.76E-03
NDUFS3	-0.482	3.19E-03	-0.334	1.10E-02
EIF2S1	-0.488	3.20E-03	-0.328	2.99E-03
DYRK2	-0.509	7.99E-03	-0.310	1.59E-02
LTA4H	-0.389	1.65E-02	-0.431	9.34E-05
SF3A3	-0.409	3.17E-02	-0.411	1.54E-05
SIGMAR1	-0.446	1.63E-02	-0.376	8.76E-04
MRTO4	-0.454	7.32E-03	-0.369	5.54E-03
UTP20	-0.422	3.38E-02	-0.400	1.21E-03
KLHL21	-0.481	4.10E-03	-0.345	3.14E-02
LTV1	-0.506	1.65E-02	-0.322	1.99E-02
NOP56	-0.421	6.21E-03	-0.408	1.23E-04
SNRPG	-0.463	8.99E-03	-0.368	2.14E-02
DYNC2H1	-0.435	7.27E-03	-0.397	8.45E-03
TIAM1	-0.535	4.17E-03	-0.298	1.39E-02

TRMT1	-0.457	1.77E-02	-0.377	1.62E-02
GNL3	-0.411	3.86E-02	-0.426	4.44E-05
CRIP2	-0.539	1.63E-02	-0.302	4.21E-02
ADGRA3	-0.510	3.01E-03	-0.332	6.14E-03
NSUN2	-0.460	6.00E-03	-0.384	7.60E-05
RNF8	-0.475	2.14E-02	-0.372	3.98E-02
ACTL6A	-0.469	1.00E-02	-0.379	2.10E-04
MRPS35	-0.491	1.89E-03	-0.359	1.07E-02
NUDC	-0.472	1.34E-03	-0.379	1.82E-04
NAT8L	-0.432	4.68E-02	-0.419	3.90E-03
GIPC1	-0.384	3.01E-02	-0.469	1.43E-03
RPS19	-0.564	2.13E-04	-0.294	9.13E-03
TTYH3	-0.546	5.51E-04	-0.313	1.83E-03
TSR1	-0.520	2.87E-03	-0.345	1.97E-03
NCR3LG1	-0.501	6.10E-03	-0.367	1.88E-02
CRKL	-0.468	2.62E-03	-0.402	4.77E-05
PFAS	-0.426	2.11E-02	-0.443	2.79E-05
MCM4	-0.440	5.54E-03	-0.432	1.04E-05
ARFGAP2	-0.574	1.52E-04	-0.298	3.67E-02
NAT10	-0.478	2.63E-03	-0.397	8.81E-04
RAC3	-0.413	3.76E-02	-0.463	1.03E-02
RPL37A	-0.586	9.84E-05	-0.290	1.52E-02
SLIRP	-0.500	3.55E-03	-0.378	2.17E-02
KCTD10	-0.528	1.46E-02	-0.350	2.53E-03
PAIP1	-0.510	1.16E-03	-0.368	1.92E-03
DDX54	-0.476	4.79E-03	-0.403	1.88E-03
IPO5	-0.453	1.81E-03	-0.428	3.31E-06
NAE1	-0.556	1.21E-03	-0.325	5.22E-03
DSTN	-0.440	1.33E-02	-0.445	4.85E-04
MRPL14	-0.496	3.70E-02	-0.390	1.61E-02
DHX37	-0.443	1.03E-02	-0.444	6.83E-04
PA2G4	-0.456	3.16E-03	-0.432	2.77E-05
SSBP1	-0.501	2.98E-03	-0.388	2.90E-03
NME1-NME2	-0.428	2.48E-02	-0.462	2.49E-06
ENOSF1	-0.476	3.36E-02	-0.415	1.13E-03
CACYBP	-0.587	1.38E-04	-0.306	1.43E-02
AC005841.1	-0.470	8.23E-03	-0.423	2.79E-02
STARD7-AS1	-0.524	9.91E-03	-0.371	4.88E-02
CDC25A	-0.512	1.46E-03	-0.383	4.43E-03
SINHCAF	-0.477	6.73E-03	-0.421	1.74E-05
CCDC86	-0.475	1.63E-02	-0.422	3.75E-03
EIF4A3	-0.529	1.31E-03	-0.371	9.05E-04
NPM1	-0.475	4.55E-03	-0.426	7.67E-07
CLTA	-0.564	7.00E-05	-0.337	5.56E-03
NOP2	-0.489	2.25E-03	-0.413	1.74E-04
PRPF40A	-0.589	4.12E-04	-0.314	4.70E-03
NOC2L	-0.523	2.90E-03	-0.380	4.80E-04
STIP1	-0.548	4.14E-05	-0.359	5.51E-05
PDCD11	-0.591	1.87E-05	-0.316	7.55E-03
PPM1G	-0.522	9.31E-04	-0.386	1.59E-04
TGIF2	-0.488	4.16E-03	-0.421	6.21E-03

UTP18	-0.550	2.84E-04	-0.359	1.23E-02
PRMT5	-0.488	4.88E-03	-0.422	1.45E-04
DHX33	-0.500	1.48E-02	-0.410	2.28E-04
PPP2R2A	-0.533	2.32E-03	-0.380	6.90E-04
PPIA	-0.450	1.90E-03	-0.463	2.21E-08
CD59	-0.603	2.18E-05	-0.310	4.56E-02
SEH1L	-0.475	1.87E-02	-0.444	1.32E-03
LYPLA2	-0.438	1.05E-02	-0.484	1.76E-04
PRMT5-AS1	-0.515	5.49E-03	-0.407	7.51E-03
MTCH2	-0.589	1.73E-05	-0.334	3.33E-03
MYL12A	-0.468	5.95E-03	-0.455	1.82E-04
PARK7	-0.480	1.70E-03	-0.445	6.69E-05
FBL	-0.459	2.55E-02	-0.467	2.24E-05
WDR4	-0.517	1.33E-02	-0.411	1.14E-02
MRPS26	-0.536	1.35E-02	-0.393	3.10E-02
TNPO2	-0.403	3.77E-02	-0.527	1.09E-08
WDR3	-0.577	3.57E-04	-0.358	4.43E-03
PODXL2	-0.486	1.71E-02	-0.449	3.87E-03
URB2	-0.501	2.09E-03	-0.434	1.83E-03
RRP1B	-0.462	1.61E-03	-0.474	1.76E-06
EIF5A	-0.425	6.89E-03	-0.511	1.02E-09
RPF2	-0.555	1.12E-03	-0.382	1.10E-02
TRIT1	-0.523	4.96E-03	-0.416	3.88E-03
DNAJC11	-0.572	2.04E-04	-0.370	7.67E-03
ATIC	-0.444	3.09E-02	-0.502	1.21E-07
HSPE1-MOB4	-0.650	5.02E-05	-0.297	1.95E-02
EBNA1BP2	-0.460	7.32E-03	-0.489	9.44E-06
CENPV	-0.601	5.32E-03	-0.350	1.96E-03
AC012640.4	-0.594	1.91E-05	-0.358	4.74E-03
PPAN-P2RY11	-0.483	1.77E-02	-0.471	4.81E-04
CCT5	-0.506	9.11E-04	-0.449	3.37E-08
CCDC138	-0.489	6.04E-03	-0.466	2.21E-03
PSMD8	-0.430	8.27E-03	-0.528	9.06E-07
C3orf52	-0.520	2.30E-02	-0.438	2.82E-02
MTHFD1	-0.426	1.48E-02	-0.533	1.69E-07
CCT8	-0.536	6.45E-04	-0.424	1.10E-06
NOC3L	-0.592	1.45E-04	-0.369	3.35E-02
SNRPB	-0.571	8.30E-04	-0.390	6.31E-04
RPS6KA1	-0.451	1.09E-02	-0.510	4.76E-03
JPT1	-0.484	7.99E-04	-0.478	5.11E-06
LDHB	-0.516	1.01E-03	-0.446	5.13E-08
GEMIN5	-0.612	8.18E-05	-0.350	1.17E-02
AKAP11	-0.634	1.64E-04	-0.335	1.68E-02
ENOPH1	-0.599	1.96E-04	-0.375	5.82E-03
AHSA1	-0.591	1.86E-04	-0.384	1.75E-03
NR1H2	-0.443	4.29E-02	-0.533	1.73E-04
CCT6A	-0.544	9.56E-05	-0.431	3.50E-06
MRPL39	-0.617	9.64E-05	-0.359	3.67E-02
SLC5A6	-0.503	4.76E-03	-0.477	1.62E-05
STOML2	-0.594	8.58E-04	-0.387	2.77E-03
STARD7	-0.643	1.22E-06	-0.339	3.07E-04

VARS	-0.461	7.54E-03	-0.525	3.89E-08
ADSL	-0.619	2.38E-05	-0.369	7.73E-04
NCL	-0.541	5.11E-04	-0.450	1.21E-07
HDAC3	-0.549	5.99E-04	-0.442	1.61E-05
HSPE1	-0.632	1.57E-04	-0.359	1.64E-03
CCT2	-0.596	1.08E-04	-0.398	8.97E-06
RFC1	-0.649	2.48E-04	-0.346	3.79E-03
SRPK1	-0.611	1.20E-05	-0.387	1.18E-04
NOP14	-0.507	3.31E-03	-0.498	1.68E-05
KAT2A	-0.499	2.56E-02	-0.508	6.95E-04
PPAN	-0.517	5.61E-03	-0.491	2.09E-04
ACP1	-0.699	3.42E-07	-0.309	2.00E-02
NDRG3	-0.596	2.88E-03	-0.416	6.56E-03
SNRPE	-0.511	8.44E-04	-0.501	2.72E-05
NPM3	-0.600	4.92E-04	-0.413	4.66E-02
PAQR4	-0.528	9.56E-03	-0.488	2.16E-03
ILF3	-0.591	8.27E-05	-0.424	1.10E-07
KATNB1	-0.615	3.65E-04	-0.402	6.08E-03
TBRG4	-0.667	1.76E-06	-0.351	2.87E-03
PPID	-0.600	2.38E-04	-0.422	2.45E-03
NACC2	-0.651	4.61E-05	-0.371	1.30E-03
EIF3B	-0.542	6.21E-04	-0.487	8.37E-10
TNFRSF21	-0.459	3.78E-02	-0.573	4.89E-04
POLR1C	-0.619	1.90E-04	-0.413	4.34E-03
SFT2D3	-0.435	3.39E-02	-0.600	6.20E-07
AIMP2	-0.607	2.83E-05	-0.429	4.01E-04
ENAH	-0.630	1.05E-05	-0.407	1.01E-05
HNRNPF	-0.553	4.78E-04	-0.485	6.43E-07
PES1	-0.517	4.16E-03	-0.524	6.41E-07
BZW2	-0.460	4.49E-03	-0.581	1.18E-07
UTP3	-0.683	6.86E-04	-0.360	3.04E-02
TMEM126B	-0.652	1.86E-04	-0.390	1.42E-02
WDR43	-0.645	3.34E-05	-0.397	7.73E-04
AIFM1	-0.654	8.09E-05	-0.393	1.36E-04
TRAP1	-0.580	5.52E-05	-0.469	1.31E-06
FKBP4	-0.514	1.90E-04	-0.538	2.32E-07
PSMG1	-0.540	8.63E-04	-0.516	1.92E-04
FBXO45	-0.719	2.61E-05	-0.344	1.39E-02
SNRPD1	-0.585	2.66E-04	-0.479	1.30E-05
HAUS7	-0.626	8.20E-04	-0.442	7.43E-03
FAM117A	-0.689	1.55E-03	-0.382	1.94E-02
LIN28B	-0.759	5.95E-06	-0.313	1.11E-02
ARMC6	-0.570	1.21E-03	-0.507	7.58E-05
BAG1	-0.629	1.01E-03	-0.455	1.06E-02
TREX2	-0.635	8.84E-04	-0.456	5.93E-03
ATP1B3	-0.703	6.77E-04	-0.393	5.13E-05
HSPD1	-0.665	7.57E-07	-0.431	1.51E-07
BRSK2	-0.636	2.20E-04	-0.461	1.20E-03
OAZ1	-0.755	6.82E-08	-0.347	3.01E-03
KDM1A	-0.608	1.49E-05	-0.504	2.18E-07
QTRT1	-0.665	1.31E-03	-0.451	1.07E-02

WDR35	-0.757	1.55E-05	-0.361	1.40E-02
PUS7	-0.700	1.21E-04	-0.417	7.46E-03
NRIP3	-0.766	3.35E-04	-0.352	2.64E-02
NME1	-0.573	2.92E-03	-0.547	4.47E-07
NELL2	-0.786	5.68E-05	-0.336	1.52E-02
HSP90AA1	-0.704	2.23E-08	-0.420	7.00E-08
RBM15B	-0.679	1.85E-05	-0.450	2.95E-05
RASL10B	-0.692	5.97E-05	-0.438	1.68E-02
AL358113.1	-0.760	2.68E-05	-0.383	6.94E-03
CNOT9	-0.604	2.78E-05	-0.539	4.05E-08
TYMS	-0.567	3.32E-02	-0.583	7.04E-08
GART	-0.699	2.82E-07	-0.451	4.03E-06
RAN	-0.579	3.35E-05	-0.572	2.60E-11
GID8	-0.864	1.03E-08	-0.291	4.18E-02
PRMT1	-0.630	6.91E-05	-0.526	6.72E-09
C1QBP	-0.595	5.20E-04	-0.562	7.28E-09
RRP1	-0.728	1.12E-05	-0.429	2.94E-03
APEX1	-0.589	5.37E-05	-0.569	4.80E-11
PAICS	-0.667	5.70E-07	-0.492	1.20E-08
RPL7L1	-0.608	1.74E-05	-0.557	8.99E-09
JAM3	-0.642	1.12E-05	-0.528	6.61E-09
AP1G1	-0.841	3.32E-10	-0.337	1.86E-03
PAWR	-0.672	1.38E-06	-0.515	2.99E-05
GRWD1	-0.842	1.68E-07	-0.354	3.87E-02
TJP2	-0.786	1.49E-05	-0.414	3.02E-03
POLR1D	-0.830	4.06E-07	-0.378	1.21E-02
PRKACA	-0.618	2.92E-03	-0.591	1.64E-07
CCT3	-0.669	9.72E-07	-0.549	1.36E-10
BID	-0.595	2.64E-03	-0.633	6.90E-05
PUF60	-0.895	4.99E-08	-0.334	1.63E-03
PGAM5	-0.698	2.14E-06	-0.533	1.27E-05
NADK	-0.811	3.03E-07	-0.421	1.10E-03
NOLC1	-0.616	1.31E-05	-0.624	1.12E-11
NLN	-0.666	1.53E-04	-0.579	9.23E-07
PPM1E	-0.873	8.50E-06	-0.375	3.95E-02
NDUFB11	-0.814	2.42E-07	-0.435	4.59E-03
UCK2	-0.710	8.22E-07	-0.545	9.59E-07
DARS	-0.832	5.96E-10	-0.442	2.25E-05
NKAIN1	-0.713	1.36E-02	-0.563	5.73E-04
UQCRFS1	-0.838	2.21E-10	-0.441	5.30E-04
XPO5	-0.838	2.40E-10	-0.443	1.62E-05
MYCN	-0.595	1.34E-03	-0.688	8.02E-12
NIFK	-0.765	5.91E-08	-0.518	2.66E-06
DSTYK	-0.906	1.09E-09	-0.378	2.16E-02
PDK1	-0.884	6.08E-07	-0.404	3.15E-02
TSN	-0.567	1.66E-04	-0.727	5.91E-15
MEX3B	-0.721	9.00E-03	-0.575	6.83E-05
RCOR2	-0.907	1.50E-06	-0.401	5.07E-03
CDC34	-0.797	3.85E-08	-0.517	1.57E-06
HSP90AB1	-0.710	1.24E-07	-0.607	2.98E-14
DCTPP1	-0.712	7.91E-05	-0.605	6.35E-06

TTLL12	-0.743	3.40E-06	-0.583	1.21E-07
CCT7	-0.871	3.39E-11	-0.467	4.87E-08
ERGIC1	-0.830	9.29E-10	-0.513	1.26E-06
SAP18	-0.775	5.95E-07	-0.580	1.69E-07
BOP1	-0.619	1.16E-04	-0.739	2.60E-10
CALM2	-1.061	2.52E-17	-0.299	2.42E-03
HNRNPH2	-0.837	4.21E-08	-0.530	3.87E-08
SBK1	-0.757	5.76E-03	-0.610	2.29E-05
CHN1	-1.012	1.38E-05	-0.360	6.46E-03
WDR12	-0.866	1.91E-09	-0.510	1.05E-05
PPA1	-0.697	1.52E-06	-0.681	5.64E-12
CAPRIN1	-0.710	3.85E-08	-0.680	1.29E-18
CISD1	-0.982	1.75E-09	-0.416	2.72E-02
UNG	-0.801	5.98E-06	-0.602	1.67E-07
MIPEP	-0.986	5.09E-08	-0.418	1.58E-02
RRP9	-0.881	1.03E-07	-0.532	3.21E-04
MED28	-0.961	2.22E-10	-0.454	1.60E-04
THYN1	-0.890	1.41E-09	-0.544	1.12E-04
MRPL21	-0.958	9.04E-10	-0.479	7.65E-03
TIMM50	-0.909	6.76E-10	-0.532	1.29E-05
KCNQ2	-1.017	7.83E-05	-0.425	2.89E-03
ABCF2	-0.989	9.17E-13	-0.455	2.55E-04
AC107081.2	-0.981	9.63E-08	-0.482	2.42E-03
DDX6	-1.196	2.36E-17	-0.271	1.01E-02
CTSC	-0.852	1.15E-07	-0.620	1.58E-07
CDC25B	-0.940	1.42E-07	-0.534	9.72E-06
POP1	-0.924	1.34E-08	-0.564	6.34E-05
PAK4	-0.827	3.48E-05	-0.682	9.59E-07
ALYREF	-0.731	9.46E-06	-0.782	5.53E-14
SAR1A	-1.081	4.79E-14	-0.441	1.09E-05
NFYB	-1.017	4.95E-11	-0.521	6.56E-04
GDAP1	-1.037	2.25E-12	-0.502	1.50E-03
GABARAPL2	-1.149	1.19E-12	-0.413	6.46E-03
MAT2B	-1.127	5.00E-17	-0.436	1.40E-05
ANKRD13B	-0.821	1.29E-04	-0.747	1.01E-07
TMEM198	-0.895	4.60E-06	-0.674	3.90E-05
DDX21	-1.025	3.32E-10	-0.554	8.44E-12
TMED4	-1.320	4.10E-24	-0.291	2.46E-02
KIAA1147	-0.982	3.54E-07	-0.629	4.28E-07
CCT4	-1.007	1.41E-14	-0.613	1.04E-13
SSX2IP	-0.982	4.16E-11	-0.640	6.58E-07
LAMP1	-1.335	5.46E-22	-0.304	7.11E-03
SEC11A	-0.957	7.87E-11	-0.704	5.53E-14
MTAP	-1.059	2.70E-10	-0.612	1.42E-08
RAB3C	-1.278	6.83E-08	-0.419	2.01E-02
EIF5A2	-1.327	1.99E-13	-0.376	2.11E-02
GTPBP4	-1.163	3.33E-14	-0.554	1.58E-07
NOL11	-1.195	1.54E-14	-0.608	1.44E-08
FAM220A	-1.006	2.36E-09	-0.825	1.14E-08
COQ5	-1.394	3.62E-16	-0.469	1.22E-02
AL391650.1	-1.396	1.24E-13	-0.510	4.70E-03

VGF	-1.508	9.80E-29	-0.399	2.73E-02
RAB39A	-1.565	1.86E-14	-0.400	2.95E-02
ZNF593	-1.450	7.83E-15	-0.540	2.29E-03
SUPT16H	-1.326	5.31E-20	-0.704	8.81E-17
SLC18B1	-1.576	4.19E-18	-0.510	6.18E-03
RRN3	-1.537	1.06E-25	-0.578	2.51E-07
NUS1	-1.525	4.93E-17	-0.635	2.90E-09
STMN3	-1.513	1.39E-23	-0.811	5.34E-12
ARMT1	-1.501	2.58E-21	-0.981	2.17E-16
RPL7A	-1.521	4.02E-28	-0.964	1.26E-40
WDR77	-1.750	1.27E-38	-0.767	1.50E-12
AL390195.1	-1.817	6.91E-38	-0.703	6.05E-07
DKC1	-1.645	1.01E-28	-1.084	2.33E-34
SLC6A15	-2.415	0.00E+00	-0.652	1.08E-06
AC008914.1	-2.092	1.26E-41	-0.985	3.25E-10
TOMM20	-2.157	0.00E+00	-1.135	1.40E-45
IL6ST	-2.257	0.00E+00	-1.537	0.00E+00

Table S4. Variant analysis of Wilms' tumour candidate genes in Wit49 and 17.94.

Genes	Wit49	17.94
CTNNB1	none	none
DICER1	none	none
DROSHA	none	none
MYCN	none	none
REST	**769P/L** (rs3796529)	none
SIX1	none	none
SIX2	none	none
TP53	**72P/R** (rs1042522, CM961374, COSV53098660); **248R/Q** (rs11540652, CM920675, COSV52661580)	**72 P/R** (rs1042522, CM961374, COSV53098660); **245G/S** (rs28934575, CM900210, COSV52661744)
TRIM28	none	none
TRIP13	none	none
WT1	none	none
WTX	none	none

Table S5. Gene Ontology analysis of MYCN-regulated genes in Wilms' tumour.

GO	Description	Enrichment	FDR	Genes
GO:0042565	RNA nuclear export complex	35.48	0.035	*XPO5,RAN*
GO:0045025	mitochondrial degradosome	35.48	0.035	*SUPV3L1,PNPT1*
GO:1904851	positive regulation of establishment of protein localization to telomere	32.36	0.000	*CCT7,CCT6A,CCT5,CCT8,CCT3,CCT2,TCP1,DKC1,CCT4*
GO:0000463	maturation of LSU-rRNA from tricistronic rRNA transcript	25.12	0.000	*WDR12,RPL7,PES1,GTPBP4,RPF2,RPL7L1,BOP1*
GO:0006189	'de novo' IMP biosynthetic process	30.20	0.000	*ATIC,PFAS,GART,PAICS,ADSL*
GO:0034709	methylosome	20.89	0.000	*WDR77,SNRPG,SNRPD1,PRMT5,SNRPE,PRMT1,SNRPB*
GO:0000466	maturation of 5.8S rRNA from tricistronic rRNA transcript	10.72	0.002	*WDR12,UTP20,PES1,PDCD11,NOP14,BOP1*
GO:0005687	U4 snRNP	15.85	0.005	*SNRPG,SNRPD1,SNRPE,SNRPB*
GO:0005683	U7 snRNP	15.49	0.023	*SNRPG,SNRPE,SNRPB*
GO:0005677	chromatin silencing complex	12.02	0.033	*SIRT2,TNRC18,BAZ2A*
GO:0070584	mitochondrion morphogenesis	8.91	0.011	*SUPV3L1,PNPT1,SSBP1,SLIRP,POLDIP2*

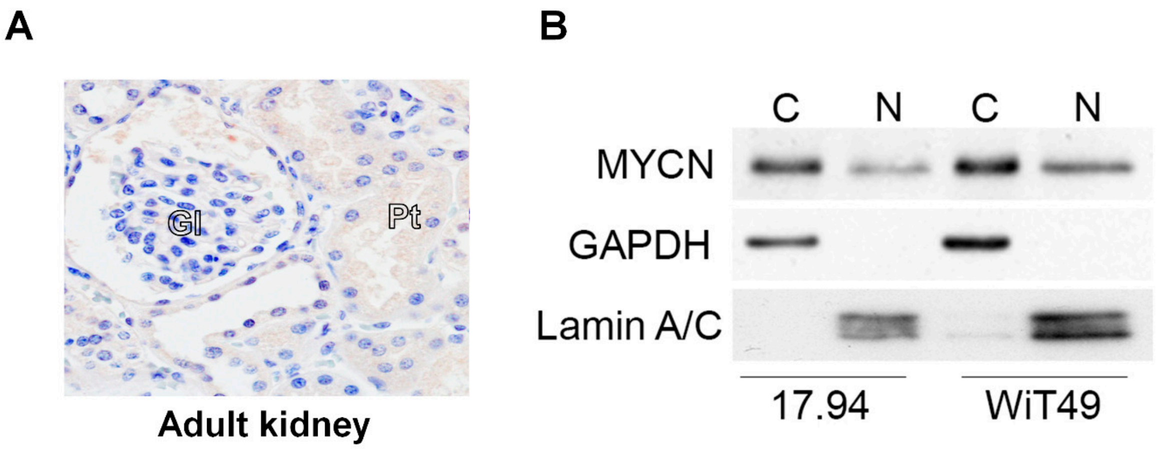

Figure S1. MYCN protein expression. (A) MYCN immunohistochemistry in an adult human kidney. (B) Western blot of MYCN protein in nuclear (N) and cytoplasmic (C) fractions in Wit49 and 17.94. Uncropped Western blots are available in Figure S5E.

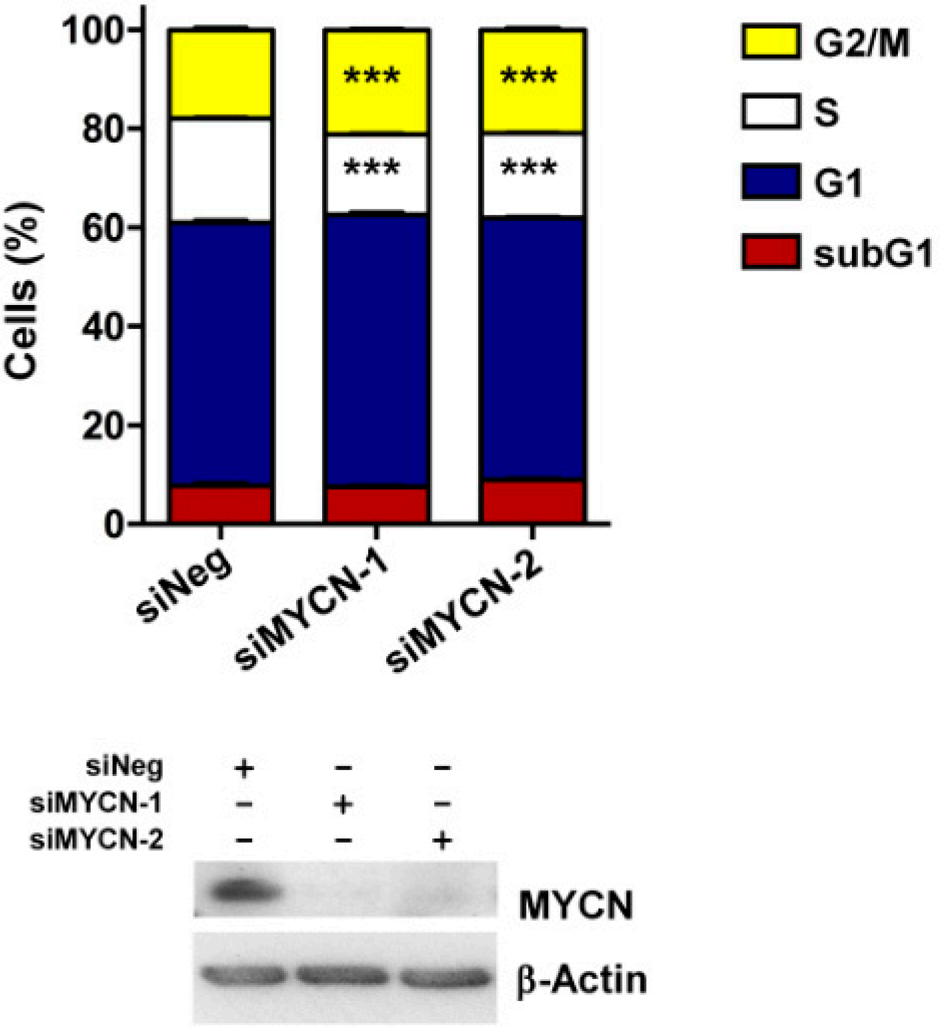

Figure S2. Cell cycle analysis. Depletion of MYCN protein in Wit49 cells by two different siRNAs for 72 hours resulted in a significant increase in the proportion of cells in G2/M phase and a decrease in S phase (n = 3, *** p < 0.01, t-test). No change was observed in the subG1 fraction. MYCN knock-down was confirmed by using Western blot. MYCN knock-down for Wit49 is also shown in Figure 7A. Uncropped Western blots are available in Figure S5F.

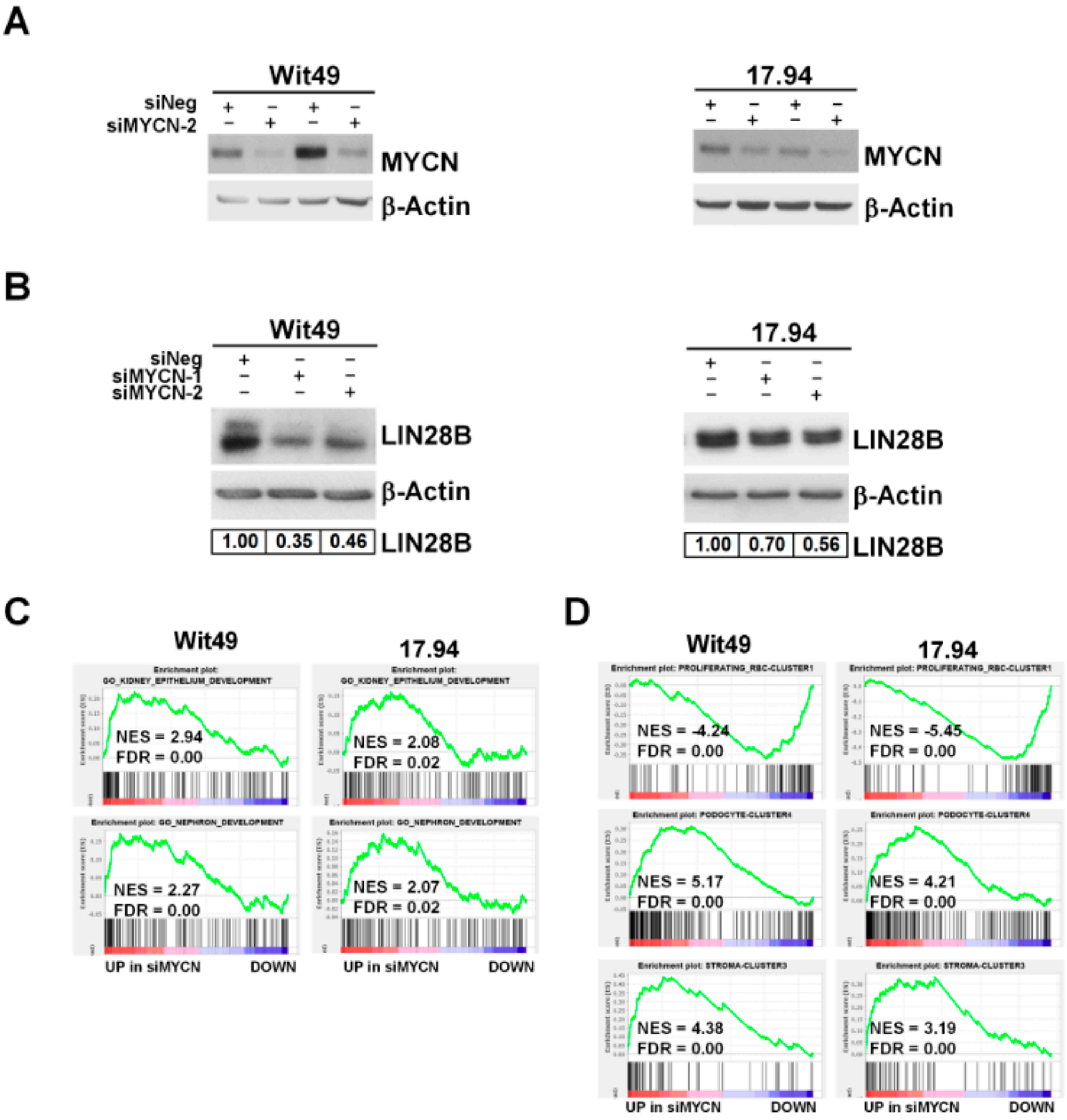

Figure S3. Western blots of MYCN and LIN28B and GSEA of differentiation gene signatures in WT. (**A**) Confirmation of MYCN depletion in samples used for RNA-seq. (**B**) Western blot showing downregulation of LIN28B protein after MYCN depletion for 72H in WT cells. Blots of confirmation of MYCN knock-down are shown in Figure 7A for Wit49 and Figure 5D for 17.94. (**C**) GSEA showed upregulation of kidney developmental gene sets in MYCN-depleted WT cells. (**D**) Single cell signatures of differentiated cell types in the fetal kidney were upregulated, while genes associated with proliferating cells were down in MYCN knock-down WT cells. Uncropped Western blots are available in Figure S5GH.

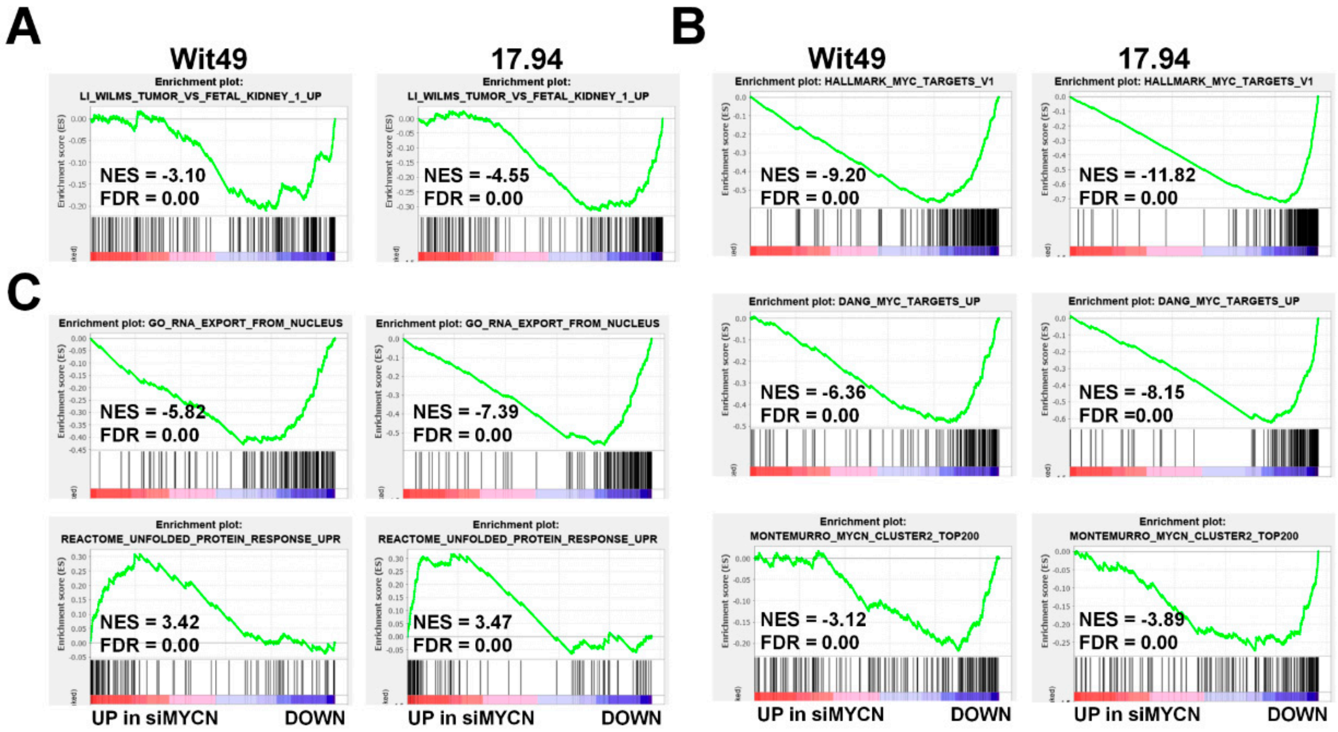

Figure S4. GSEA analysis in transcriptomes of MYCN-depleted WT cells. (**A**) Genes overexpressed in WT vs. fetal kidney were mostly downregulated. (**B**) GSEA showed downregulation of MYC/MYCN gene sets. (**C**) GSEA highlighted downregulation of gene sets associated with RNA export from the nucleus and upregulation of unfolded protein response genes.

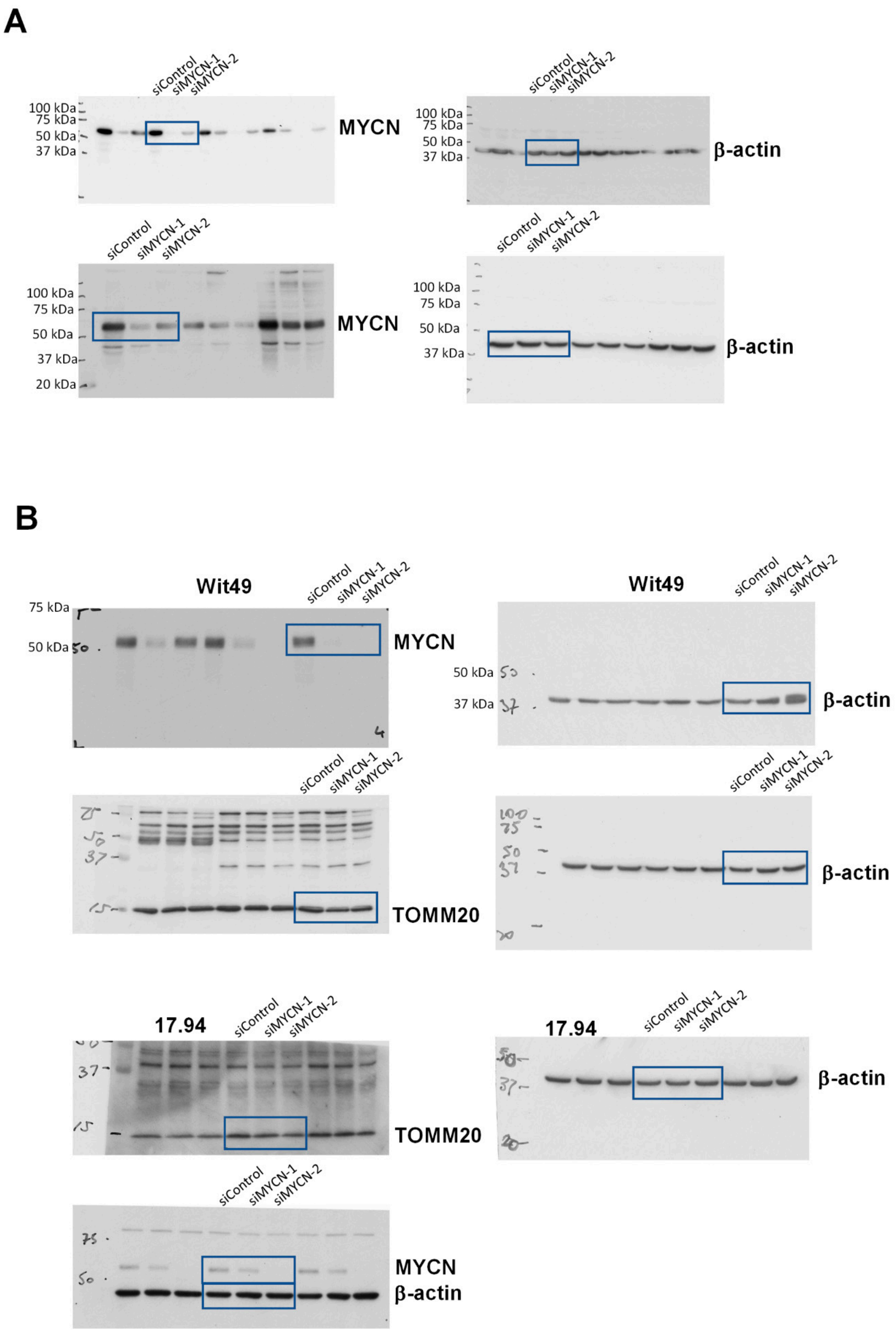
A
siControl
siMYCN-1
siMYCN-2
100 kDa
75 kDa
50 kDa
37 kDa
MYCN
β-actin
siControl
siMYCN-1
siMYCN-2
100 kDa
75 kDa
50 kDa
37 kDa
20 kDa
MYCN
β-actin
B
Wit49
siControl
siMYCN-1
siMYCN-2
75 kDa
50 kDa
MYCN
Wit49
50 kDa
37 kDa
β-actin
TOMM20
β-actin
17.94
TOMM20
17.94
β-actin
MYCN
β-actin

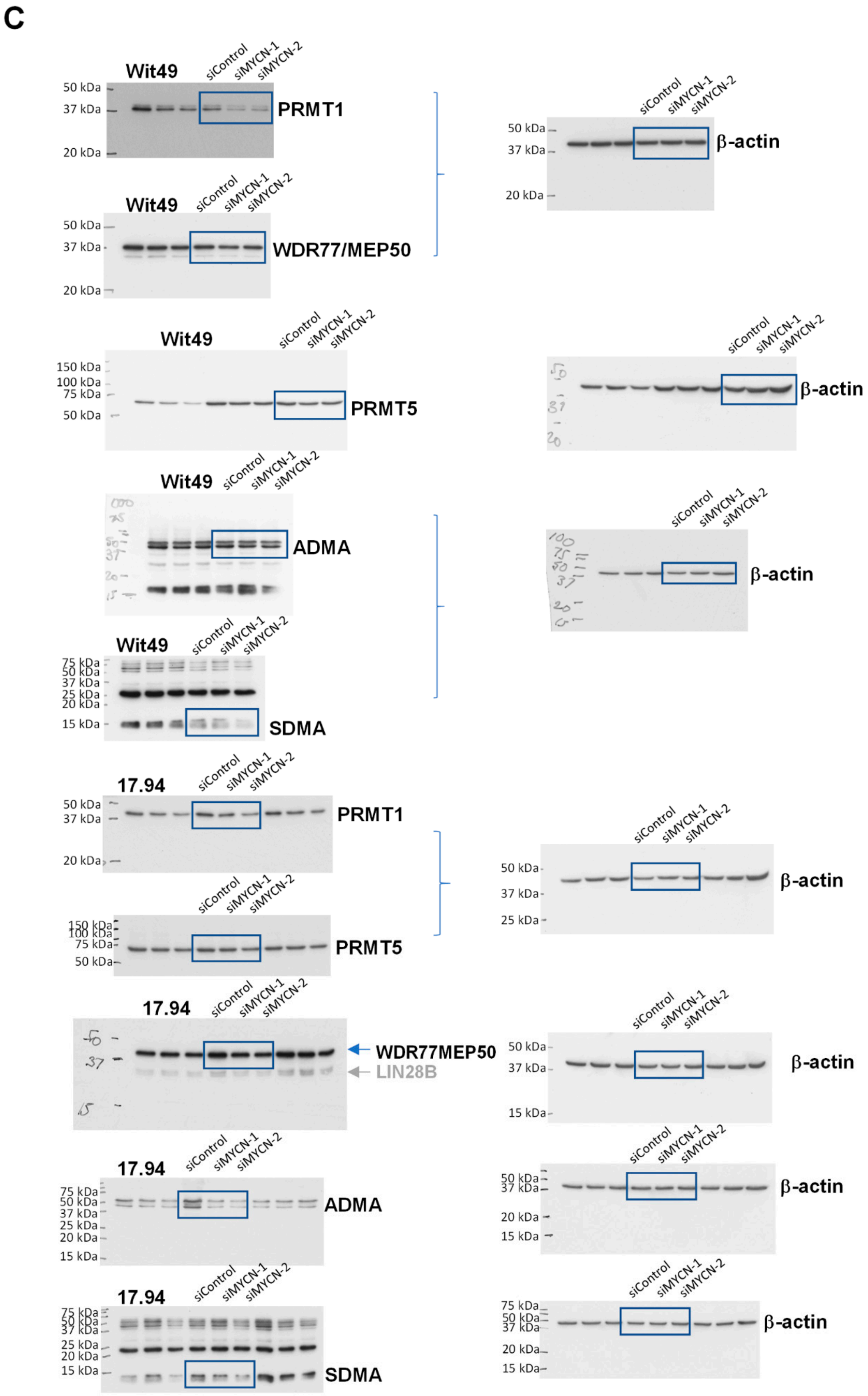
C
Wit49
siControl
siMYCN-1
siMYCN-2
50 kDa
37 kDa
20 kDa
PRMT1
WDR77/MEP50
β-actin
150 kDa
100 kDa
75 kDa
PRMT5
ADMA
SDMA
25 kDa
15 kDa
17.94
WDR77MEP50
LIN28B

D.

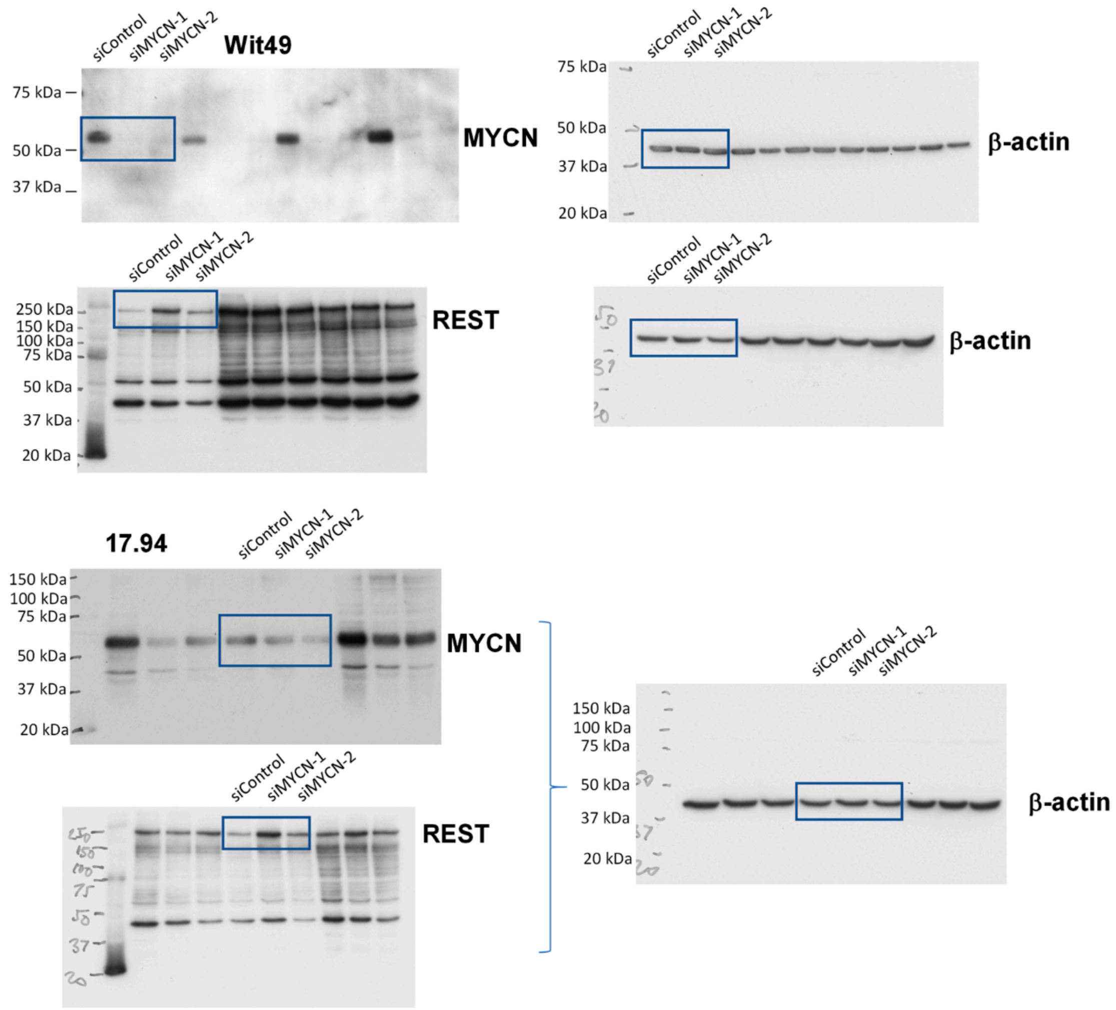
siControl
siMYCN-1
siMYCN-2
Wit49
75 kDa
50 kDa
37 kDa
MYCN
75 kDa
50 kDa
37 kDa
20 kDa
β-actin
250 kDa
150 kDa
100 kDa
75 kDa
50 kDa
37 kDa
20 kDa
REST
β-actin
17.94
150 kDa
100 kDa
75 kDa
50 kDa
37 kDa
20 kDa
MYCN
REST
150 kDa
100 kDa
75 kDa
50 kDa
37 kDa
20 kDa
β-actin

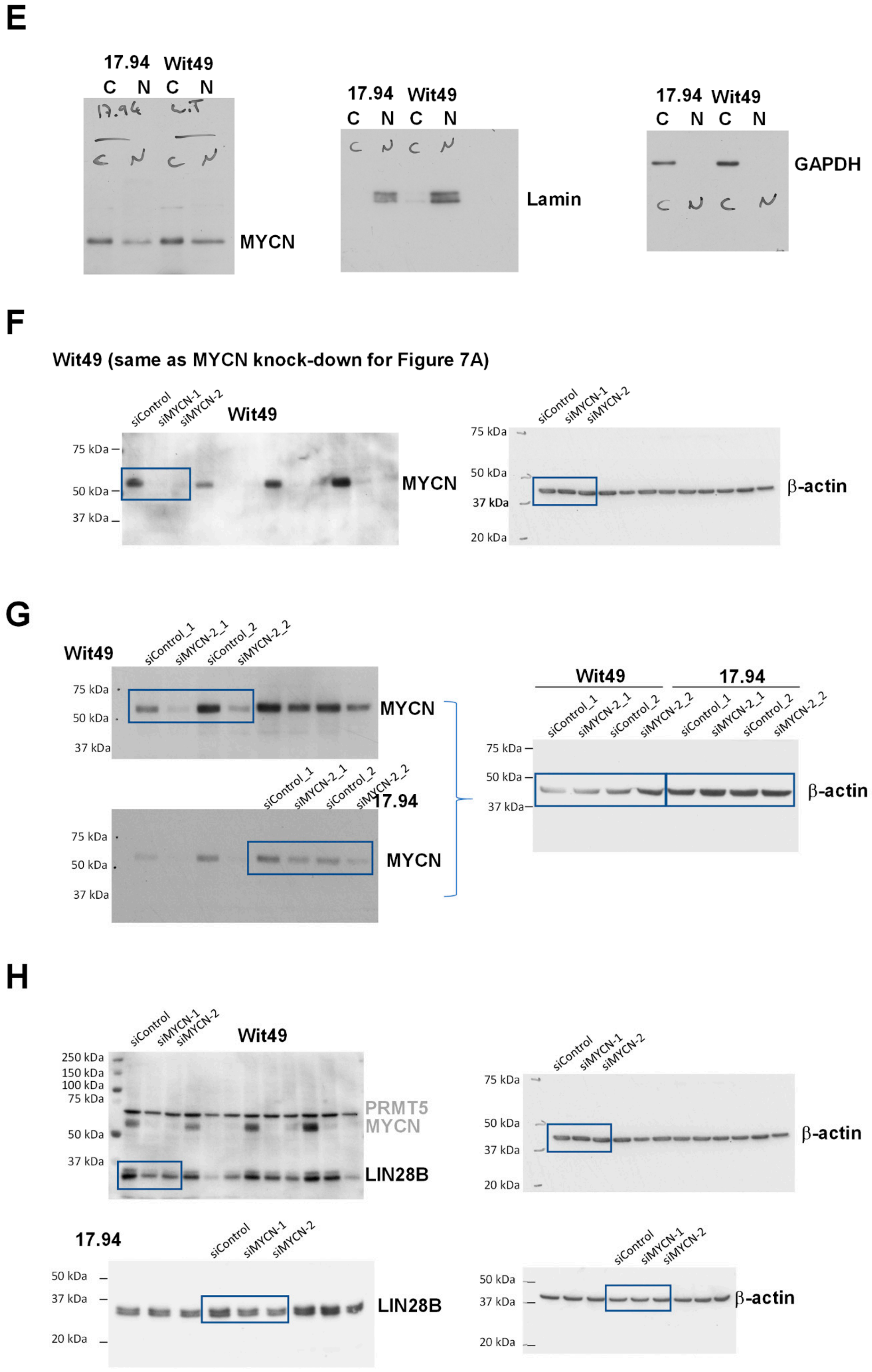
E
17.94 Wit49
C N C N
MYCN
17.94 Wit49
C N C N
Lamin
17.94 Wit49
C N C N
GAPDH
F
Wit49 (same as MYCN knock-down for Figure 7A)
siControl siMYCN-1 siMYCN-2
Wit49
75 kDa
50 kDa
37 kDa
MYCN
siControl siMYCN-1 siMYCN-2
75 kDa
50 kDa
37 kDa
20 kDa
β-actin
G
Wit49
siControl_1 siMYCN-2_1 siControl_2 siMYCN-2_2
75 kDa
50 kDa
37 kDa
MYCN
siControl_1 siMYCN-2_1 siControl_2 siMYCN-2_2
17.94
75 kDa
50 kDa
37 kDa
MYCN
Wit49
17.94
siControl_1 siMYCN-2_1 siControl_2 siMYCN-2_2 siControl_1 siMYCN-2_1 siControl_2 siMYCN-2_2
75 kDa
50 kDa
37 kDa
β-actin
H
siControl siMYCN-1 siMYCN-2
Wit49
250 kDa
150 kDa
100 kDa
75 kDa
50 kDa
37 kDa
PRMT5
MYCN
LIN28B
siControl siMYCN-1 siMYCN-2
75 kDa
50 kDa
37 kDa
20 kDa
β-actin
17.94
siControl siMYCN-1 siMYCN-2
50 kDa
37 kDa
20 kDa
LIN28B
siControl siMYCN-1 siMYCN-2
50 kDa
37 kDa
20 kDa
β-actin

Figure S5. Uncropped images of Western blots. (**A**) Uncropped images of Western blots in Figure 2A and B. (**B**) Uncropped images of blots in Figure 5D. (**C**) Uncropped images of blots in Figure 6C. (**D**) Uncropped images of blots in Figure 7A. (**E**) Uncropped images of blots in Figure S1B. (**F**) Uncropped images of blots in Figure S2. (**G**) Uncropped images of blots in Figure S3A. (**H**) Uncropped images of blots in Figure S3B.
